# Phase Equilibrium Calculations of Solid–Liquid Quaternary System Na_2_CO_3_-Na_2_SO_4_-H_2_O_2_-H_2_O at 5 °C

**DOI:** 10.3390/molecules31091497

**Published:** 2026-04-30

**Authors:** Guo-En Li, Fan Shi, Yue Liu, Yu-Long Li

**Affiliations:** 1 Department of Environmental and Chemical Engineering, Hebei Vocational University of Industry and Technology, Shijiazhuang 050091, China; liguoen1987@163.com (G.-E.L.); 18931883137@163.com (F.S.); 13933828837@163.com (Y.-L.L.); 2 Department of Chemical and Environmental Engineering, Hebei Chemical & Pharmaceutical Vocational Technology College, Shijiazhuang 050026, China

**Keywords:** red mud, sodium percarbonate, sodium sulfate, Pitzer model, phase diagram

## Abstract

Red mud discharged during alumina production via the Bayer process is characterized by high contents of sodium carbonate, sodium sulfate, and other soluble salts, and it remains poorly utilized and accumulates in long-term stockpiles. Sodium percarbonate has found extensive industrial applications, and its synthesis via the salting-out method represents one of the dominant industrial routes. In this context, sodium sulfate was employed as a salting-out agent. On the basis of relevant ternary systems, the phase equilibrium of the quaternary system Na_2_CO_3_–Na_2_SO_4_–H_2_O_2_–H_2_O at 5 °C was systematically investigated and calculated. The objective was to utilize red mud as a waste resource and develop a novel integrated process that favored the wet synthesis of sodium percarbonate while enabling the efficient separation of sodium salts. The solubility data for the ternary subsystems constituting the above quaternary system were correlated using the Pitzer model, yielding the corresponding ion interaction parameters and activity coefficients. The validated model was then applied to predict the phase equilibrium data of the quaternary system. Verification results indicate that the calculated values are in satisfactory agreement with the experimental data. On the basis of the phase equilibrium data of the Na_2_CO_3_–Na_2_SO_4_–H_2_O_2_–H_2_O system at 5 °C, a phase diagram was constructed. Along with five solid-phase crystallization fields, three invariant points were identified: the co-saturation point of Na_2_SO_4_·10H_2_O, Na_2_CO_3_·10H_2_O, and Na_2_CO_3_·1.5H_2_O_2_·H_2_O; the co-saturation point of Na_2_SO_4_·10H_2_O, Na_2_CO_3_·1.5H_2_O_2_·H_2_O, and Na_2_SO_4_·0.5H_2_O_2_·H_2_O; and the co-saturation point of Na_2_CO_3_·1.5H_2_O_2_·H_2_O, Na_2_SO_4_·0.5H_2_O_2_·H_2_O, and Na_2_CO_3_·2H_2_O_2_·H_2_O. From phase diagram analysis, a novel wet process route for sodium percarbonate production using waste red mud is proposed. The process involves chemical reaction, crystallization, separation, and drying to obtain the final product. A new process flow diagram for the value-added production of sodium percarbonate is also presented.

## 1. Introduction

In the technological process of alumina production via the Bayer process, various carbonate minerals (CaCO_3_, CaCO_3_·MgCO_3_) are present in the raw materials (bauxite, lime, and alkali) [1,2,3,4,5,6,7,8]. These minerals tend to decompose during the digestion process, which can convert sodium hydroxide in the production process into sodium carbonate. Meanwhile, due to the influence of air during the stirring process, sodium aluminate in the solution is also prone to acidification by CO_2_ in the air, resulting in the generation of a large amount of sodium carbonate. Owing to the high content of sulfur in bauxite and coupled with the use of materials such as alkali and water, the concentration of sodium sulfate in the solution continuously increases [9,10,11,12,13]. During the evaporation of mother liquor, the concentrations of sodium carbonate and sodium sulfate increase rapidly and precipitate in a crystalline state, leading to the accumulation of a large amount of sodium salt crystals. These sodium salt crystals are mainly discharged along with red mud; therefore, a large amount of sodium carbonate and sodium sulfate in red mud cannot be rationally utilized and separated, which has become a common problem faced by alumina production via the Bayer process [14,15].

Given the high content of Na_2_CO_3_ in red mud, the synthesis of sodium percarbonate has become a viable option for its rational utilization [16,17,18]. Sodium percarbonate is a widely used inorganic fine chemical product, which has found extensive applications in fields such as the washing of synthetic fibers, disinfection of tableware and food, preservation of fruits, and sewage treatment [19,20,21]. Its production methods include dry and wet processes, among which the wet process is currently the most commonly used industrial production method. The wet synthesis of sodium percarbonate is achieved by combining solid Na_2_CO_3_ with a certain concentration of H_2_O_2_. It is non-toxic and does not cause any irritation under diluted conditions, thus posing no pollution to the environment in practical applications. Sodium sulfate, also known as anhydrous Glauber’s salt, is a white, odorless, and slightly bitter powder or crystal. In organic synthesis laboratories, sodium sulfate is a commonly used post-treatment desiccant and is also often used to remove barium salts [22,23]. In chemical production, it can also be used in the manufacture of sodium sulfide, sodium silicate, and other products. Taking advantage of the significant difference in solubility between sodium percarbonate and Na_2_SO_4_, there is a difference in the sequence of crystallization precipitation, thereby realizing the separation of Na_2_SO_4_ and significantly reducing the H_2_O_2_ content in the mother liquor [8,24,25].

The Na_2_SO_4_ salting-out method is an effective approach to improve the traditional wet synthesis process of sodium percarbonate, and its theoretical basis is the phase diagram of the Na_2_CO_3_-Na_2_SO_4_-H_2_O_2_-H_2_O quaternary system. At present, Guo-en Li and Yue Liu et al. have conducted research on the phase equilibrium of this quaternary system [17,18]. For the purpose of better guiding the practical production design through the solubility of this system, this paper calculates the phase equilibrium of the Na_2_CO_3_-Na_2_SO_4_-H_2_O_2_-H_2_O quaternary system.

## 2. Derivation of the Pitzer Model

### 2.1. Selection of the Model

The Pitzer electrolyte solution activity model and its modified models are the most widely used theoretical models for electrolyte solutions nowadays. The calculation formulas of this model can be applied to more than 280 types of high-concentration electrolyte solutions, including the calculation of mixed electrolytes. In the Na_2_CO_3_-Na_2_SO_4_-H_2_O_2_-H_2_O quaternary system studied in this paper, H_2_O_2_ has an extremely small ionization constant and can be regarded as a non-electrolyte. Therefore, this quaternary system is an electrolyte–electrolyte–non-electrolyte–water system, and thus this model can be selected for the phase equilibrium calculation in this paper.

### 2.2. Calculation Expression of Activity Coefficient

Assuming that the solution contains *n_w_* kilograms of water as well as moles *n_i_* and *n_j_* of solute ions *i* and *j*, the expression for the total excess Gibbs energy in this system is as follows:

(1) $$\begin{array}{ll} \frac{G^{E}}{RT} & ={\left(\frac{G^{E}}{RT}\right)}_{i-i}+{\left(\frac{G^{E}}{RT}\right)}_{A-i}+{\left(\frac{G^{E}}{RT}\right)}_{A-A}\\ & =n_{w}f(I)+\frac{1}{n_{w}}\sum_{i}\sum_{j}\lambda_{ij}(I)n_{i}n_{j}+\frac{1}{n_{w}^{2}}\sum_{i}\sum_{j}\sum_{k}\mu_{ijk}n_{i}n_{j}n_{k}\\ & +\frac{1}{n_{w}}\sum_{i}\theta_{Ai}n_{A}n_{i}+\frac{1}{n_{w}^{2}}\sum_{i}\sum_{j}\psi_{Aij}\left(I\right)n_{A}n_{i}n_{j}+\frac{1}{n_{w}}\lambda_{AA}n_{A}^{2}+\frac{1}{n_{w}^{2}}\mu_{AAA}n_{A}^{3} \end{array}$$

In the above equation:

$\frac{G^{E}}{RT}$—the total excess Gibbs energy of the solution;
${\left(\frac{G^{E}}{RT}\right)}_{i-i}$—the increment of excess Gibbs energy caused by the interactions between ions in the solution;
${\left(\frac{G^{E}}{RT}\right)}_{i-A}$—the increment of excess Gibbs energy caused by the interactions between ions and non-electrolyte molecules in the solution;
${\left(\frac{G^{E}}{RT}\right)}_{A-A}$—the increment of excess Gibbs energy caused by the interactions between non-electrolyte molecules in the solution.

By taking the partial derivatives of Equation (1) with respect to *n_i_* and *n_A_*, the general formulas for the activity coefficients of ion i and non-electrolyte molecule *A* in any electrolyte–non-electrolyte–water system can be obtained. Since the system studied in this paper is an electrolyte–electrolyte–non-electrolyte–water system, only the simplified form of the Pitzer equation suitable for this system is derived here [26,27,28,29].

We assume that the studied system is *MX*-*MY*-*A*-H_2_O, where *MX* and *MY* are a pair of electrolytes with a common cation. We let *ν_M_* and *ν_X_* as well as *ν’_M_* and *ν_Y_* be the numbers of cations and anions per molecule of *MX* and *MY*, respectively, while *A* denotes the non-electrolyte. By letting the molalities of *MX* and *MY* be m_MX_ and m_MY_, respectively, then it can be obtained that:

(2) $$\left.\begin{array}{l} m_{M}=\nu_{M}m_{MX}+\nu \prime_{M}m_{MY}\\ m_{X}=\nu_{X}m_{MX}\\ m_{Y}=\nu_{Y}m_{MY} \end{array}\right\}$$

We take the partial derivatives of Equation (1) with respect to *n_M_* and *n_X_*, multiply the obtained results by *ν_M_* and *ν_X_* correspondingly, then sum them up, and substitute Equation (2) into Equation (1). Then we let:

$$B_{MX}=\beta_{MX}^{\left(0\right)}+\frac{2\beta_{MX}^{\left(1\right)}}{\alpha^{2}I}\left[1-\left(1+\alpha I^{\frac{1}{2}}\right)\exp\left(-\alpha I^{\frac{1}{2}}\right)\right]$$

$$B_{MX}^{\prime }=\frac{2\beta_{MX}^{\left(1\right)}}{\alpha^{2}I^{2}}\left[-1+\left(1+\alpha I^{\frac{1}{2}}+\frac{1}{2}\alpha^{2}I\right)\exp\left(-\alpha I^{\frac{1}{2}}\right)\right]$$

$$C_{MX}=\frac{C_{MX}^{\phi }}{2{\left|Z_{M}Z_{X}\right|}^{\frac{1}{2}}}$$

In the above equation, $\beta_{\mathrm{MX}}^{\left(0\right)}$, $\beta_{\mathrm{MX}}^{\left(1\right)}$, and $C_{\mathrm{MX}}^{\left(\varphi \right)}$ are the virial coefficients of the electrolyte *MX*; thus, we obtain:

(3) $$\begin{array}{ll} \ln\gamma_{\pm MX} & =\frac{1}{2}\left|Z_{M}Z_{X}\right|f\prime \left(I\right)+\left(\frac{2\nu_{M}}{\nu }\right)\sum_{a}m_{a}\left[B_{Ma}+\left(\sum mz\right)C_{Ma}\right]\\ & +\left(\frac{2\nu_{X}}{\nu }\right)\sum_{c}m_{c}\left[B_{cX}+\left(\sum mz\right)C_{cX}\right]+\sum_{c}\sum_{a}m_{c}m_{a}\left[\left|Z_{M}Z_{X}\right|B\prime_{ca}+\frac{2\nu_{M}Z_{M}}{\nu }C_{ca}\right]\\ & +\left(\frac{2\nu_{X}}{\nu }\right)\sum_{a}m_{a}\theta_{Xa}\left(I\right)+\sum_{c}\sum_{a}m_{c}m_{a}\cdot \frac{1}{\nu }\left(\nu_{M}\psi_{Mca}+\nu_{X}\psi_{caX}\right)\\ & +\frac{1}{2}\sum_{a}\sum_{a\prime }m_{a}m_{a\prime }\left[\left(\frac{\nu_{M}}{\nu }\right)\psi_{Maa\prime }+\left|Z_{M}Z_{X}\right|\theta \prime_{aa\prime }\left(I\right)\right]+\frac{m_{A}}{\nu }\left(\nu_{M}\theta_{AM}+\nu_{X}\theta_{AX}\right)\\ & +\frac{1}{\nu }\left[2\nu_{M}\sum_{j}\psi_{AMj}\left(I\right)+2\nu_{X}\sum_{j}\psi_{AXj}\left(I\right)\right]m_{A}m_{j}\\ & +\frac{Z_{M}^{2}\nu_{M}}{2\nu }\sum_{j}\sum_{k}\psi \prime_{Akj}m_{A}m_{j}m_{k}+\frac{Z_{X}^{2}\nu_{X}}{2\nu }\sum_{j}\sum_{k}\psi \prime_{Akj}m_{A}m_{j}m_{k} \end{array}$$

By taking the partial derivative of Equation (1) with respect to *n_A_*, the expression for the activity coefficient of the non-electrolyte *A* is as follows:

(4) $$\ln\gamma_{A}=2\lambda_{AA}m_{A}+3\mu_{AAA}m_{A}^{2}+\sum_{i}\theta_{Ai}m_{i}+\sum_{i}\sum_{j}\psi_{Aij}m_{i}m_{j}$$

Here, *λ_AA_* and *μ_AAA_* are the two-particle and three-particle interaction coefficients between H_2_O_2_ molecules, respectively. In the equation for $\sum \mathrm{mz}=\sum_{a}m_{a}|z_{a}|=\sum_{c}m_{c}z_{c}$, the subscript c denotes the cation and a denotes the anion, with *ν* = *ν_M_* + *ν_X_*.

In the above equation, *i*, *j*, and *k* include all ions while excluding non-electrolyte molecules; *θ* and *λ* are the two-particle interaction parameters; *μ* and *Ψ* are the three-particle interaction parameters; *Ψ_Aij_(I)* is a function of ionic strength; F(I) is the intensity function of ions; and *λ_AA_*, *μ_AAA_*, *θ_Ai_*, and *μ_ijk_* are assumed to be independent of ionic strength, as follows:

(5) $$\psi_{Aij}(I)={\psi^{\left(0\right)}}_{Aij}+F(I){\psi^{\left(1\right)}}_{Aij}$$

(6) $$F\left(I\right)=\frac{2}{\alpha^{2}I}\left[1-\left(1+\alpha I^{0.5}\right)\exp\left(-\alpha I^{0.5}\right)\right]\;\left(\alpha =2\right)$$

Here, *f(I)* is the long-range electrostatic interaction term in the Pitzer model; by taking the partial derivatives of the above equation with respect to *n_i_* and *n_A_* to obtain *f’(I)*, the general formulas for the activity coefficients of ion i and non-electrolyte molecule *A* in any non-electrolyte–electrolyte–electrolyte–water system can be obtained. From this, the expressions for the activity coefficients of each substance in the system studied in this paper can be derived as follows:

(7) $$\begin{array}{ll} \ln\gamma_{Na_{2}CO_{3}} & =f^{\prime }\left(I\right)+\frac{2}{3}\left(m_{\mathrm{Na}}+2m_{CO_{3}}\right)\left[B_{Na_{2}CO_{3}}+\left(\sum mz\right)C_{Na_{2}CO_{3}}\right]\\ & +\frac{4}{3}m_{SO_{4}}\left[B_{{\mathrm{Na}}_{2}SO_{4}}+\left(\sum mz\right)C_{{\mathrm{Na}}_{2}SO_{4}}\right]+m_{\mathrm{Na}}m_{SO_{4}}\left(2{B_{{\mathrm{Na}}_{2}SO_{4}}}^{\prime }+\frac{4}{3}C_{{\mathrm{Na}}_{2}SO_{4}}\right)\\ & +m_{\mathrm{Na}}m_{CO_{3}}\left(2{B_{Na_{2}CO_{3}}}^{\prime }+\frac{4}{3}C_{Na_{2}CO_{3}}\right)+\frac{2}{3}m_{SO_{4}}\left[\theta_{SO_{4},CO_{3}}^{\left(0\right)}+F\left(I\right){\theta_{SO_{4}}}_{,CO_{3}}^{\left(1\right)}\right]\\ & +2m_{SO_{4}}m_{CO_{3}}F^{\prime }\left(I\right)\theta_{SO_{4},CO_{3}}^{\left(1\right)}+\frac{1}{3}\left(2m_{CO_{3}}m_{SO_{4}}+m_{\mathrm{Na}}m_{SO_{4}}\right)\psi_{\mathrm{Na},SO_{4},CO_{3}}\\ & +\frac{1}{3}m_{A}\theta_{\mathrm{Na},CO_{3},A}+\frac{4}{3}m_{A}m_{CO_{3}}\left[\psi_{\mathrm{Na},CO_{3},A}^{\left(0\right)}+F\left(I\right)\psi_{\mathrm{Na},CO_{3},A}^{\left(1\right)}\right]\\ & \frac{4}{3}m_{A}m_{SO_{4}}\left[\psi_{\mathrm{Na},SO_{4},A}^{\left(0\right)}+F\left(I\right)\psi_{\mathrm{Na},SO_{4},A}^{\left(1\right)}\right]+\frac{4}{3}m_{A}m_{\mathrm{Na}}\left[\psi_{\mathrm{Na},CO_{3},A}^{\left(0\right)}+F\left(I\right)\psi_{\mathrm{Na},CO_{3},A}^{\left(1\right)}\right]\\ & +2m_{A}m_{\mathrm{Na}}m_{SO_{4}}F^{\prime }\left(I\right)\psi_{\mathrm{Na},SO_{4},A}^{\left(1\right)}+2m_{A}m_{\mathrm{Na}}m_{CO_{3}}F^{\prime }\left(I\right)\psi_{\mathrm{Na},CO_{3},A}^{\left(1\right)} \end{array}$$

(8) $$\begin{array}{ll} \ln\gamma_{N{\mathrm{Na}}_{2}SO_{4}} & =f^{\prime }\left(I\right)+\frac{2}{3}\left(m_{\mathrm{Na}}+2m_{SO_{4}}\right)\left[B_{Na_{2}SO_{4}}+\left(\sum mz\right)C_{Na_{2}SO_{4}}\right]\\ & +\frac{4}{3}m_{CO_{3}}\left[B_{{\mathrm{Na}}_{2}CO_{3}}+\left(\sum mz\right)C_{{\mathrm{Na}}_{2}CO_{3}}\right]+m_{\mathrm{Na}}m_{CO_{3}}\left(2{B_{{\mathrm{Na}}_{2}{CO}_{3}}}^{\prime }+\frac{4}{3}C_{{\mathrm{Na}}_{2}{CO}_{3}}\right)\\ & +m_{\mathrm{Na}}m_{SO_{4}}\left(2{B_{Na_{2}SO_{4}}}^{\prime }+\frac{4}{3}C_{Na_{2}SO_{4}}\right)+\frac{2}{3}m_{CO_{3}}\left[\theta_{CO_{3},SO_{4}}^{\left(0\right)}+F\left(I\right)\theta_{CO_{3},SO_{4}}^{\left(1\right)}\right]\\ & +2m_{SO_{4}}m_{CO_{3}}F^{\prime }\left(I\right)\theta_{SO_{4},CO_{3}}^{\left(1\right)}+\frac{1}{3}\left(2m_{CO_{3}}m_{SO_{4}}+m_{\mathrm{Na}}m_{CO_{3}}\right)\psi_{\mathrm{Na},SO_{4},CO_{3}}\\ & +\frac{1}{3}m_{A}\theta_{\mathrm{Na},SO_{4},A}+\frac{4}{3}m_{A}m_{CO_{3}}\left[\psi_{\mathrm{Na},SO_{4},A}^{\left(0\right)}+F\left(I\right)\psi_{\mathrm{Na},SO_{4},A}^{\left(1\right)}\right]\\ & \frac{4}{3}m_{A}m_{CO_{3}}\left[\psi_{\mathrm{Na},CO_{3},A}^{\left(0\right)}+F\left(I\right)\psi_{\mathrm{Na},CO_{3},A}^{\left(1\right)}\right]+\frac{4}{3}m_{A}m_{\mathrm{Na}}\left[\psi_{\mathrm{Na},SO_{4},A}^{\left(0\right)}+F\left(I\right)\psi_{\mathrm{Na},SO_{4},A}^{\left(1\right)}\right]\\ & +2m_{A}m_{\mathrm{Na}}m_{CO_{3}}F^{\prime }\left(I\right)\psi_{\mathrm{Na},CO_{3},A}^{\left(1\right)}+2m_{A}m_{\mathrm{Na}}m_{SO_{4}}F^{\prime }\left(I\right)\psi_{\mathrm{Na},SO_{4},A}^{\left(1\right)} \end{array}$$

(9) $$\begin{array}{ll} \ln\gamma_{H_{2}O_{2}} & =m_{SO_{4}}\theta_{\mathrm{Na},SO_{4},A}+m_{CO_{3}}\theta_{\mathrm{Na},CO_{3},A}+2m_{\mathrm{Na}}m_{CO_{3}}\left[\psi_{\mathrm{Na},CO_{3},A}^{\left(0\right)}+F\left(I\right)\psi_{\mathrm{Na},CO_{3},A}^{\left(1\right)}\right]\\ & +2m_{\mathrm{Na}}m_{SO_{4}}\left[\psi_{\mathrm{Na},SO_{4},A}^{\left(0\right)}+F\left(I\right)\psi_{\mathrm{Na},SO_{4},A}^{\left(1\right)}\right]+2m_{A}\lambda_{AA}+3m_{A}^{2}\mu_{AAA} \end{array}$$

### 2.3. Calculation of Water Activity

The activity of water *a_H_*_2*O*_ is calculated from the osmotic coefficient as follows:

(10) $$\ln a_{H_{2}O}=-\frac{\sum_{i}m_{i}}{55.51}\varphi$$

In the above equation, *m_i_* is the molality of ion *i*, ∑mi denotes the summation over all ions except non-electrolytes, and 55.51 is the molar number of 1000 g of water.

The osmotic coefficient is obtained from the following equation:

(11) $$\phi -1=\frac{1}{\sum_{i}m_{i}}\left\{\left[If^{\prime }\left(I\right)-f\left(I\right)\right]+2\sum_{c}\sum_{a}m_{c}m_{a}\left[B_{ca}+{{IB}^{\prime }}_{ca}+2\left(\sum mz\right)C_{ca}\right]\right\}$$

Using Equations (7)–(11), the activity coefficients of electrolytes *MX*, *MY*, and non-electrolyte *A* in the liquid phase of the *MX*-*MY*-*A*-H_2_O system, as well as the activity of solvent H_2_O, can be calculated. According to the principle that when the system reaches liquid–solid phase equilibrium—the activity of the liquid phase that precipitates the same solid phase must be equal—calculations can be carried out. For example, by calculating equation $a_{Na2SO4\cdot 10H2O}=m_{Na2SO4}\gamma_{Na2SO4}{a_{H2O}}^{10}$, the activity expression of solid phase Na_2_SO_4_·10H_2_O can be calculated; by calculating equation $a_{Na2CO3\cdot 1.5H2O2\cdot H2O}=m_{Na2CO3}\gamma_{Na2CO3}{m_{H2O2}}^{1.5}{\gamma_{H2O2}}^{1.5}a_{H2O}$, the activity expression of solid phase Na_2_CO_3_·1.5H_2_O_2_·H_2_O can be calculated; and by calculating equation $a_{Na2CO3\cdot 10H2O}=m_{Na2CO3}\gamma_{Na2CO3}{a_{H2O}}^{10}$, the activity expression of solid phase Na_2_CO_3_·10H_2_O can be calculated. Similar methods are used in other situations, so we will not list them one by one here. Furthermore, the solubility of each salt in the liquid phase can be calculated and correlated.

## 3. Results and Discussion

### 3.1. Model Parameters

The activity coefficient of H_2_O_2_ at 5 °C is not available in the literature. In this study, the molecular interaction parameters in the activity coefficient model were treated as unknowns and regressed from the solubility data, which is consistent with the derivation requirements of the Pitzer model. Moreover, the correlation results indicate that this approach is indeed feasible. The Pitzer parameters of Na_2_CO_3_ and Na_2_SO_4_ at 25 °C, the temperature-dependent analytical expressions of the parameters, and the relational expressions of the parameters with temperature have been reported in the literature [29].

(12) $$X\left(T\right)=X_{Tr}+{\left(\frac{\partial X}{\partial T}\right)}_{T=Tr}(T-T_{r})+\frac{1}{2}{\left(\frac{\partial^{2}X}{\partial T^{2}}\right)}_{T=Tr}{\left(T-T_{r}\right)}^{2}\;\left(T_{r}=298.15\;K\right)$$

Based on the above equation, the Pitzer parameters of Na_2_CO_3_ and Na_2_SO_4_ at 5 °C were thus calculated, and the calculated values are presented in Table 1.

### 3.2. Parameter Correlation for Phase Equilibrium Calculation of Ternary Subsystems

Based on the phase equilibrium data of the ternary systems Na_2_CO_3_-H_2_O_2_-H_2_O, Na_2_SO_4_-H_2_O_2_-H_2_O, and Na_2_CO_3_-Na_2_SO_4_-H_2_O at 5 °C, the interaction parameters of the ternary systems were regressed, and the results are presented in Table 2.

### 3.3. Prediction of Solubility of Quaternary System

In the quaternary system, five solid phases precipitate at 5 °C, namely Na_2_CO_3_·1.5H_2_O_2_·H_2_O, Na_2_CO_3_·10H_2_O, Na_2_SO_4_·10H_2_O, Na_2_CO_3_·2H_2_O_2_·10H_2_O, and Na_2_SO_4_·0.5H_2_O_2_·H_2_O, with no double salts formed. According to the principle that the activity of the same solid phase precipitated from the quaternary system must be equal to that when the salt is saturated alone, the activity coefficient model was applied to predict the solubility of the Na_2_CO_3_-Na_2_SO_4_-H_2_O_2_-H_2_O quaternary system at 5 °C. The calculated results and their comparison with the experimental values [18] are presented in Table 3.

Data in Table 3 indicate that the solubility data of the quaternary system calculated by the regressed parameters are basically consistent with the experimental values. This indicates that it is feasible to use the Pitzer model to calculate the liquid–solid phase equilibrium of this system.

### 3.4. Analysis of Sodium Percarbonate Production Process at 5 °C

**Figure 1:**
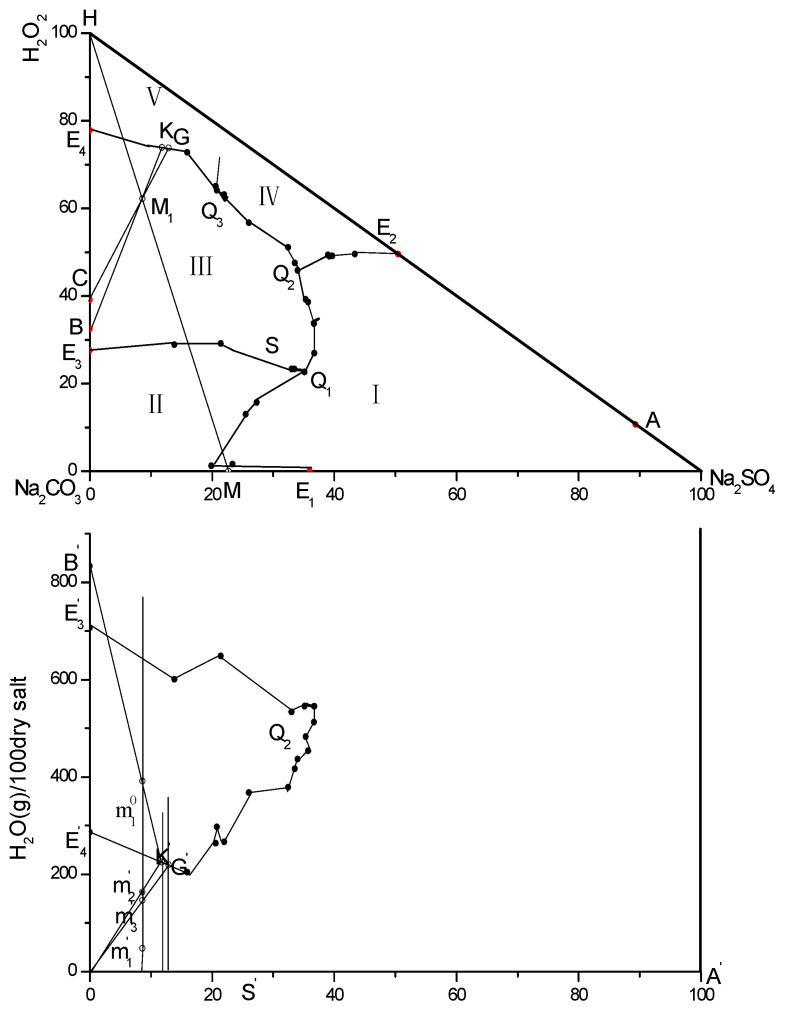
Analytical calculations of the phase diagram and water diagram for Na_2_CO_3_-Na_2_SO_4_-H_2_O_2_-H_2_O quaternary system.

Based on the data in Table 3, the phase diagram and water diagram of the Na_2_CO_3_-Na_2_SO_4_-H_2_O_2_-H_2_O quaternary system were plotted, as shown in Figure 1. It is determined that there are three eutectic points in this system, which are the eutectic points of Na_2_SO_4_·10H_2_O, Na_2_CO_3_·10H_2_O, and Na_2_CO_3_·1.5H_2_O_2_·H_2_O; the eutectic points of Na_2_SO_4_·10H_2_O, Na_2_CO_3_·1.5H_2_O_2_·H_2_O, and Na_2_SO_4_·0.5H_2_O_2_·H_2_O; and the eutectic points of Na_2_CO_3_·1.5H_2_O_2_·H_2_O, Na_2_SO_4_·0.5H_2_O_2_·H_2_O, and Na_2_CO_3_·2H_2_O_2_·H_2_O. In addition, the system contains five solid-phase crystallization regions corresponding to the above solid phases.

**Figure 2:**
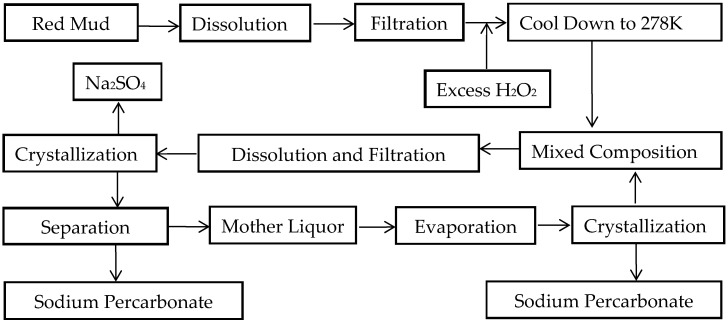
Process flowchart for the production and separation of sodium bicarbonate and sodium sulfate.

As a common theory for discussing the deposition and transformation rules of natural salts, the phase equilibrium theory of aqueous salt systems can serve as a methodological theory for determining the concentration route of the liquid phase and the sequence of salt precipitation during the evaporation or cooling process of brine, on the premise of applying phase chemistry theory and phase diagram data. Therefore, the direction and limit of a series of changes that will occur in the system when its external conditions change can be pre-analyzed through the phase diagram. By analyzing the above-mentioned phase diagram, the following production process can be formulated: first, separate pure substances from the mixed solution; second, prepare hydrates, double salts or solid solutions using single salts; and finally, explore a reasonable method for the cyclic utilization of part or all of the recovered mother liquor to improve the product recovery rate. The details are shown in Figure 2.

## 4. Conclusions

The Pitzer model was applied to regress the solubility data of the ternary sub-systems contained in the quaternary system, yielding the corresponding interparticle interaction parameters and activities. Subsequently, the established model was used to predict the solubility data of the quaternary system. Verification results indicated that the calculated data were in good agreement with the experimental data. Based on the phase equilibrium data of the Na_2_CO_3_-Na_2_SO_4_-H_2_O_2_-H_2_O quaternary system at 5 °C, a phase diagram was constructed. It was confirmed that there were three eutectic points in the system, which corresponded to the co-saturation of Na_2_SO_4_·10H_2_O, Na_2_CO_3_·10H_2_O, and Na_2_CO_3_·1.5H_2_O_2_·H_2_O; the co-saturation of Na_2_SO_4_·10H_2_O, Na_2_CO_3_·1.5H_2_O_2_·H_2_O, and Na_2_SO_4_·0.5H_2_O_2_·H_2_O; and the co-saturation of Na_2_CO_3_·1.5H_2_O_2_·H_2_O, Na_2_SO_4_·0.5H_2_O_2_·H_2_O, and Na_2_CO_3_·2H_2_O_2_·H_2_O. Additionally, five solid-phase crystallization regions were identified in the system. Through the analysis of the phase diagram, a process was proposed where sodium percarbonate product was synthesized via a wet process reaction using waste red mud as the raw material at an appropriate temperature, followed by further crystallization, separation, and drying processes to obtain the final product. A new process flow diagram for value-added sodium percarbonate chemical products was also established. This work provides a certain theoretical and technical basis for the production process of synthesizing sodium percarbonate via the wet method using waste red mud.

## Figures and Tables

**Table 1:** Virial coefficients of Na_2_CO_3_ and Na_2_SO_4_ at 5 °C.

Electrolyte	*β^(0)^*	*β^(1)^*	*C^ϕ^*
Na_2_CO_3_	0.03620	1.5101	0.00520
Na_2_SO_4_	−0.02797	0.8353	0.05607

**Table 2:** Interaction parameters of particles in ternary system at 5 °C.

System	Interaction Parameters of Particles				
Na_2_CO_3_-H_2_O_2_-H_2_O	$\lambda_{AA}$	$\mu_{AAA}$	$\theta_{\mathrm{Na},CO_{3},A}$	$\psi_{\mathrm{Na},CO_{3},A}^{\left(0\right)}$	$\psi_{\mathrm{Na},CO_{3},A}^{\left(1\right)}$
	−0.768399	0.0091285	−2.936690	−0.437680	9.342605
Na_2_SO_4_-H_2_O_2_-H_2_O			$\theta_{\mathrm{Na},SO_{4},A}$	$\psi_{\mathrm{Na},SO_{4},A}^{\left(0\right)}$	$\psi_{\mathrm{Na},SO_{4},A}^{\left(1\right)}$
			−0.015501	−0.007124	0.074320
Na_2_CO_3_-Na_2_SO_4_-H_2_O			$\theta_{SO_{4},CO_{3}}^{\left(0\right)}$	${\theta_{SO_{4}}}_{,CO_{3}}^{\left(1\right)}$	$\psi_{\mathrm{Na},SO_{4},CO_{3}}$
			0.157500	1.712401	−0.074510

**Table 3:** Calculated and measured values of solubility for Na_2_CO_3_-Na_2_SO_4_-H_2_O_2_-H_2_O system at 5 °C ^a^.

Measured Values of Equilibrium Liquid Phase Composition (wt%)			Calculated Values of Equilibrium Liquid Phase Composition (wt%)			Fitting Error (%) ^c^			Equilibrium Solid Phase
Na_2_SO_4_	H_2_O_2_	Na_2_CO_3_	Na_2_SO_4_	H_2_O_2_	Na_2_CO_3_	Na_2_SO_4_	H_2_O_2_	Na_2_CO_3_	
2.86 ^b^	3.89	6.62	2.87	3.91	93.22	0.35	0.51	0.03	N_10_ + B
3.47 ^b^	7.25	14.37	3.44	7.22	89.34	0.86	0.41	0.07	N_10_ + B
3.78 ^b^	1.92	9.13	3.71	1.95	94.34	1.85	1.56	0.04	S_10_ + N_10_
3.86 ^b^	0.24 ^b^	15.30	3.95	0.25	95.80	2.33	4.17	0.10	S_10_ + N_10_
4.53	2.61	9.46	4.56	2.53	92.91	0.66	3.07	0.05	S_10_ + N_10_
5.12	3.56	6.62	5.23	3.41	91.36	2.15	4.21	0.04	S_10_ + N_10_ + B
5.79	3.73	0.68	5.98	3.55	90.47	3.28	4.83	0.01	S_10_ + N_10_ + B
5.56	12.11	3.70	5.61	12.49	81.90	0.90	3.14	0.52	A + B
5.69	4.17	5.64	5.44	4.02	90.54	4.39	3.60	0.44	S_10_ +B
5.25	23.93	3.70	5.03	24.32	70.65	4.19	1.63	0.24	B + C
5.98	5.5	4.83	6.07	5.22	88.71	1.51	5.09	0.21	S_10_ + B
6.45	6.97	4.63	6.36	6.74	86.90	1.40	3.30	0.37	S_10_ + B
6.06	6.72	4.37	6.35	6.98	86.67	4.79	3.87	0.63	S_10_ + B
6.33	8.54	3.76	5.99	8.17	85.84	5.37	4.33	0.83	B + S_10_ + A
6.49	9.19	3.67	6.02	8.94	85.04	7.24	2.72	0.85	B + S_10_ + A
6.78	10.66	3.46	6.45	9.86	83.69	4.87	7.50	1.37	A + B
14.71	18.21	4.14	14.08	17.59	68.33	4.28	3.40	1.86	S_10_ + A
5.23	16.11	3.78	5.41	17.02	77.57	3.44	5.65	1.39	A + B + C
5.66	17.87	3.93	5.88	18.33	75.79	3.89	2.57	0.89	A + B + C
6.00	17.22	4.08	6.21	16.91	76.88	3.50	1.80	0.13	A + B + C
19.47	22.22	3.20	20.07	21.86	58.07	3.08	1.62	0.41	S_10_ +A
4.40	0.30 ^b^	14.16	4.29	0.29	95.42	2.50	3.33	0.13	S_10_ + N_10_
12.98	16.23	3.88	13.05	15.93	71.02	0.54	1.85	0.32	S_10_ + A
9.91	12.56	2.96	9.55	12.05	78.40	3.63	4.06	1.12	S_10_ + A
5.21	3.68	6.89	5.54	3.82	90.64	6.33	3.80	0.52	S_10_ + B

A: Na_2_SO_4_·0.5H_2_O_2_·H_2_O; B: Na_2_CO_3_·1.5H_2_O_2_·H_2_O; C: Na_2_CO_3_·2H_2_O_2_·H_2_O. S_10_: Na_2_SO_4_·10H_2_O; N_10_: Na_2_CO_3_·10H_2_O. ^a^ Standard uncertainty: u(T) = 0.05 °C; relative standard uncertainties: u_r_(Na_2_SO_4_ wt%) = 0.007; u_r_(Na_2_CO_3_ wt%) = 0.011; and u_r_(H_2_O_2_ wt%) = 0.004 without those labeled with superscript. The quantity wt% means mass percentage. ^b^ Relative standard uncertainties: u_r_(Na_2_SO_4_ wt%) = 0.053; u_r_(Na_2_CO_3_ wt%) = 0.052; and u_r_(H_2_O_2_ wt%) = 0.053. ^c^ Note: Fitting error (%) = |(calculated values − measured values)/measured values|.

## Data Availability

The original contributions presented in this study are included in the article. Further inquiries can be directed to the corresponding author.
